# Maternal Complications Related to Operative Vaginal Delivery and Their Associated Factors among Women Delivered at NEMCS Hospital, Southwest Ethiopia

**DOI:** 10.1155/2023/4214252

**Published:** 2023-06-16

**Authors:** Selamu Abose Nedamo, Amanuel Nuramo Sakelo, Lire Lemma Tirore, Ageze Abose Abate

**Affiliations:** ^1^Department of Midwifery, College of Medicine and Health Sciences Wachemo University, Hossana, SNNPR, Ethiopia; ^2^Department of Public Health, College of Medicine and Health Sciences, Wachemo University, Hosanna, SNNPR, Ethiopia; ^3^Department of Medical Laboratory, College of Medicine and Health Sciences, Wachemo University, Hosanna, SNNPR, Ethiopia

## Abstract

**Background:**

Operative vaginal delivery refers to vaginal delivery performed with the use of instruments such as forceps or vacuum. Operative vaginal delivery-related maternal complications are still a serious problem, but they are one of the least investigated in Ethiopia, particularly in the study area. Increased difficulties have been attributed to a lack of understanding on how to anticipate the procedure's complications. Identifying typical OVD complications can assist health providers in detecting and intervening early. The goal of this study was to find out which characteristics contributed to maternal problems during surgical vaginal birth.

**Methods:**

A health facility-based cross-sectional study design was used. From December 2019 to November 2021, a total of 326 mother's OVD medical records were selected from a total of 1000 OVD medical records using a simple random sampling method. A checklist was used to collect the data. Binary logistic regression was computed and variables with a *p* value ≤0.2 in the bivariate logistic regression were taken to multivariate logistic regression analysis to examine the real relationship or statistical association with the outcome variable. The *p* value of <0.05 with a 95% confidence interval was considered a significant variable. The results are presented using tables, figures, and texts.

**Results:**

Maternal complications were prevalent in 62 of the cases (19%). The type of operative vaginal delivery instrument used (AOR = 2.248; 95% CI (1.144, 4.416)), the station of the presenting part at which the OVD was performed (AOR = 3.199; 95% CI (1.359, 7.533)), neonatal birth weight (AOR = 3.342; 95% CI (1.435, 7.787)), and duration of the second stage (AOR = 2.556; 95% CI (1.039, 6.284)) were significantly associated with the unfavorable maternal outcomes of operative vaginal delivery.

**Conclusions:**

Maternal complications are high in the study area. The type of operative vaginal delivery used, the duration of the second stage, the station of the presenting part at which the OVD was performed, and neonatal birth weights were all significantly related to maternal complications. While using the instrument, mothers with the identified factors should be given special attention.

## 1. Introduction

Labor and childbirth are complex physiological processes involving two human beings, the mother and the baby. Operative vaginal delivery is a vaginal delivery performed with instruments such as forceps or a vacuum [1]. Forceps, vacuum delivery, manual rotation, episiotomy, and, on rare occasions, symphysiotomy can all be used for vaginal childbirth [2]. The force generated in a closed space (vacuum) can be increased to aid in the delivery of a fetus while forceps apply traction on the fetal skull's parietal and malar bones [3].

Each pregnant woman expects spontaneous vaginal childbirth with little or no requirement for surgical procedures at the end of her pregnancy. Some, however, require assistance to avoid negative outcomes for the mother and the fetus [4].

To achieve the best possible results, current global obstetric practice advocates the use of instrumental vaginal delivery procedures. Poor labor progress, maternal exhaustion, presumed fetal risk, medical issues necessitating a shortening of the second stage of labor, and other common clinical problems are indications of OVD [5]. According to the Royal College of Obstetricians and Gynecologists, forceps/vacuum-assisted vaginal delivery was used in approximately 11% of the cases in Australia [6].

It is logical that not all operative vaginal births are the same in terms of difficulty or maternal risk [7]. Numerous studies compared the use of forceps and vacuum, and the risk of maternal injury was higher in forceps groups than in vacuum groups. Serious complications are uncommon with forceps and vacuum deliveries, but they can result in long-term maternity complications [8].

Cervical tear, vaginal tear, bleeding, third and fourth degree tear of the perineum, and anal sphincter lesions are all complications of OVD that can cause serious health problems for the mother. She could be chronically ill, which could lead to divorce and the loss of financial support [9].

The increase in problems might be due to a lack of understanding of the procedure's potential complications. The ability to recognize typical OVD issues aids healthcare workers in detecting complications and intervening as soon as possible. OVD-related maternal complications are still a serious problem, but they are one of the least investigated in Ethiopia, as well as in the study area. As a result, the study's goal is to look at maternal complications and their associated factors in operational vaginal deliveries at Nigist Eleni Mohammed Memorial Compressive Specialized Hospital in Haddiya, SNNPR, Ethiopia.

## 2. Methods and Materials

### 2.1. Study Area and Period

From November 1, 2021 to December 30, 2021, a health facility-based study was conducted at Nigist Eleni Mohammed Memorial Comprehensive Specialized Hospital in the Hadiya zone, South Nation Nationality People Area, Ethiopia. Nigist Eleni Mohammed Memorial Comprehensive Specialized Hospital is found in Hosanna town, the capital of the Hadiya zone of Ethiopia's Southern Nations, Nationalities, and Peoples' Region/SNNPR/, and is located 230 kilometers from the country's capital, Addis Ababa.

### 2.2. Study Design

A health facility-based cross-sectional study design was used.

## 3. Source Population and Study Population

### 3.1. Source Population

All mothers who gave birth by forceps or vacuum at Nigist Eleni Mohammed Memorial Compressive Specialized Hospital, Hadiya zone, South Nation Nationality People Region, Ethiopia.

### 3.2. Study Population

All mothers who gave birth by forceps or vacuum at Nigist Eleni Mohammed Memorial Compressive Specialized Hospital, Hadiya zone, South Nation Nationality People Region, Ethiopia, from November 1, 2019 to December 30, 2021.

## 4. Inclusion Criteria and Exclusion Criteria

### 4.1. Inclusion Criteria

The study included all selected medical records of mothers who gave birth by forceps or vacuum-assisted delivery.

### 4.2. Exclusion Criteria

Incomplete medical records of mothers who gave birth by OVD were excluded.

## 5. Sample Size Determination and Sampling Procedure

### 5.1. Sample Size Determination

The sample size was calculated by using a single population proportion formula by taking the proportion of maternal complications of operative deliveries in Hawassa (30.6%) [10]. By assuming a 5% margin of error and 95% CI, the minimum desired sample size was calculated as *n* = (z*α*/2)^2^*P*(1 − *p*)/*d*^2^, where “*p*” is the proportion of maternal complications of operative vaginal, “*n*” is the minimum sample size, “*d*” is the degree of precision (how large error be tolerated) (5%), and “*z*_*α*/2_” is the 95% confidence interval (which is 1.96).

(1) $$\begin{array}{c} n=\frac{{\left(1.96\right)}^{2}\left(0.306\right)\;\left(1-0.306\right)}{{\left(0.05\right)}^{2}}=326\text{.} \end{array}$$

### 5.2. Sampling Procedure

A simple random sampling technique was used to select maternal medical records to be reviewed from those 1000 medical records by using their medical registrar number as the sampling frame.

## 6. Study Variables

The dependent variables include the following:

- Maternal complication of OVD

 The independent variables are as follows:

- Sociodemographic characteristics (age, marital status, residence, occupation, and religion).
- Obstetric characteristics (parity, weight of fetus, position of the fetal head, station of the fetal head, gestational age, ANC follow-up visits, health institution where ANC was attended, episiotomy, previous health institution delivery, and previous mode of delivery).
- Indications of operative vaginal delivery (prolonged 2^nd^ stage of labor, severe preeclampsia and/or eclampsia, heart disease, maternal exhaustion, severe anemia, and fetal distress),
- Procedure-related (operator of the procedure, duration of second stage, and type of instrument used).

## 7. Operational Definitions

### 7.1. Operative Vaginal Delivery

It refers to a delivery in which the operator uses forceps or a vacuum device to assist the mother in transitioning the fetus to extrauterine life during the second stage of labor.

### 7.2. Maternal Complications

Mother who developed at least one of the following maternal complications such as PPH, perianal tear such as the second-degree vaginal tear, third-degree, or fourth-degree tear, episiotomy extension, periuretheral/labial tear, cervical tear, traumatic PPH, and death.

### 7.3. Incomplete Charts

Incomplete charts were the medical records of mothers which lacked history and delivery summary.

## 8. Data Collection Tool and Procedures

### 8.1. Data Collection Tools

Closed and open-ended extraction format which is developed in the English language by reviewing pieces of literature was used to collect data. Data were collected using the pretested and structured questionnaire.

### 8.2. Data Collection Process

Four data collectors who had completed their BSc in midwifery from a recognized university and two MSc in maternity as supervisors were recruited and the hospital's chief executive officer and the head of the gynecology and obstetrics ward were met and asked for permission. The data collection was held for a total of 60 days.

### 8.3. Data Quality Control and Assurance Management

Pretest was carried out on 5% of the sample one month before the actual data collection in Durame General Hospital and the questions were revised based on the response obtained so that the questions that created ambiguity were rephrased. The data collectors and supervisors were trained for two days on techniques of sampling, data collection, and important points. Data entry and cleaning were performed in EpiData.

### 8.4. Data Analysis Procedure

The data were first entered to EpiData version 4.2 and then exported to SPSS version 20 to be cleaned and analyzed. Binary logistic regression was computed and variables with a *p* value of ≤0.2 in the bivariate logistic regression were taken to multivariate logistic regression analysis to examine the real relationship or statistical association with the outcome variable. The *p* value of <0.05 with a 95% confidence interval was considered a significant variable. The results are presented using tables, figures, and texts.

## 9. Results

### 9.1. Sociodemographic Characteristics

In this study, 326 records of mothers who had given birth via OVD were reviewed. The mean age of the mothers was 26.1 years (SD + 6.069), and 108 (33.1%) were between the ages of 20 and 24. While the minimum and maximum ages were 15 and 41 years, respectively, and 292 (89.6%) of the mothers were married. The majority of the mothers in the study (262 (80.4%)) were from urban areas. Almost half of the mothers (147 (45.1%)) were housewives. The majority of mothers, 259 (79.4%), were Orthodox Christians (Table 1).

### 9.2. Obstetric Characteristics

Of the 326 mothers' medical records, 183 (56.1%) were Para I, with 32 (17.5%) having varying levels of adverse maternal outcomes. 296 (90.8%) mothers received ANC follow-up ≥4 times. Episiotomy was performed on 201 mothers (61.7%) to facilitate interventions, 30 (14.9%) of whom were unfavorable. While 36 (15.8%) of the 228 (69.9%) mothers with a second-stage labor lasting from 1 to 2 hours experienced various types of adverse maternal outcomes. Only 33 (14.7%) of the 224 (68.7%) vacuum extraction procedures resulted in adverse maternal outcomes. The average newborn weight was 3248.48 g whereas neonatal weight ranged from 2500 to 3999 g (218 (66.9%)). The lowest and highest newborn weights were 1500 and 4567 grams, respectively (Table 2).

**Figure 1:**
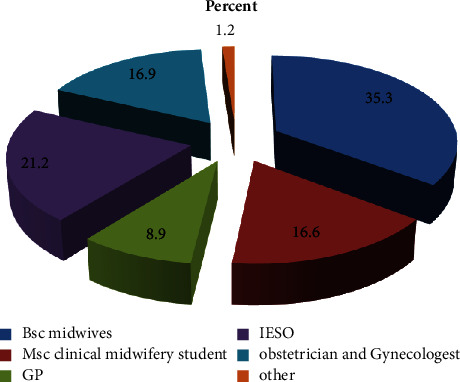
Proportion of health professionals who attended OVD at NEMMCS hospital, Hadiya zone, southwest Ethiopia, 2021 (*n* = 326).

The majority of the interventions, that is, 115 (35.3%) were conducted by BSc Midwives professionals followed by IESO with 69 students (21.2%) (Figure 1).

### 9.3. Indications for Operative Vaginal Delivery

OVD was performed in 183 (56.1%) of the 326 mother's charts due to fetal distress, followed by 89 (27.3%) delayed 2nd stage of labor (Table 3).

### 9.4. Maternal Outcome of OVD

**Figure 2:**
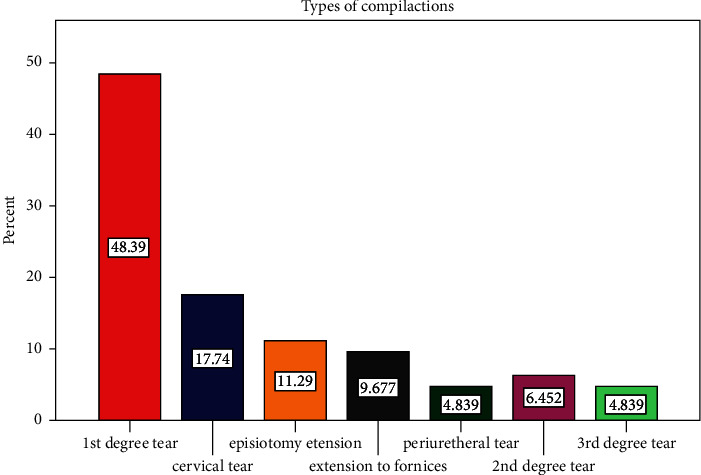
Causes of PPH among mothers, who gave birth by operative vaginal delivery OVD at NEMMCS hospital, Hadiya zone, southwest Ethiopia, 2019–2021 (*n* = 326).

Among the 326 mothers whose records were used for the study, 62 (19%) of them encountered maternal complications due to the OVD procedure. In 21 PPH cases, no maternal records of postpartum hysterectomy was found, with only three (0.9%) mothers receiving 2−3 units of blood (Figure 2).

The majority of mothers, that is, 232 (71.2%) stayed at the hospital for about 6 to 24 hours. 67 (20.6%) of the mothers stayed for 25 to 72 hours and 27 (8.3%) mothers stayed ≥73 hours. On discharge, almost all 324 (99.4%) of the mothers were free of maternal complications, but 2 (0.6%) of them had low hemoglobin levels.

### 9.5. Factors Affecting Maternal Outcome

In a bivariate analysis, variables such as place of residence, position of the fetal head, episiotomy, station during OVD application, duration of 2^nd^ stage of labor, types of operative vaginal delivery used, neonatal weight, prolonged second stage, fetal distress, and malposition were associated with maternal complications.

After fitting multivariate analyses, neonatal weight, types of OVD used, station during OVD application, and duration of the second stage were found to be significantly associated with maternal complication (Table 4).

## 10. Discussion

In this study, the extent of the mother's unfavorable outcome with different complications proved to be 19%. This finding is similar to the study conducted at Lumbini Medical College Teaching Hospital, Nepal (17.3%) [11]. The disparity could be attributed to the study design, sample size, and study area. However, this study is smaller than that conducted at Suhul General Hospital (45.4%) [15]. This variation might be sample sizes or the attendant ability to attend an OVD.

This study revealed that the most common maternal complication was a first-degree vaginal tear (48.39%), followed by a cervical tear (17.74%) and episiotomy extension (11.29%). This study was consistent with the study conducted in Hyderabad, Pakistan [16].

This study revealed a postpartum hemorrhage rate of 6.4%. This finding was consistent with the study conducted in Jimma Medical Centre (3.3%), tertiary hospital of Mumbai, India (4.01%), Lumbini Teaching Hospital of Nepal (3.8%), and Amino Kano Hospital, Nigeria (9.5%) [14, 17–19]. However, this study's result of postpartum hemorrhage rate was lower than that of the University Hospital of Port, Nigeria (42.3%) [20]. This may be due to poor documentation practice of specific hospitals.

Neonatal birth weight (≥4000 gm) was significantly associated with unfavorable maternal outcome. This result is consistent with another study conducted in Jimma [11, 19].

A low station during OVD application is significantly associated with unfavorable maternal outcomes. This might be a low station that increases the risk of injury to maternal tissue during application when compared with the midstation and outlet application of forceps or vacuum. However, the study conducted in Suhul Shire showed that midtypes of the instrument were associated with maternal complications [13]. This difference may be due to the varieties of OVD types which have been applied.

The study also revealed that forceps device types were significantly associated with an unfavorable maternal outcome. Similar results were reported at Hawassa University Teaching and Referral Hospital; Liverpool Hospital, Australia; Shankar Nagar, India; and Jinnah Hospital, Lahore [10, 12, 15]. The notion is that the operator has greater familiarity with the type of instrument, more manipulation, and issues with proper evaluation and application. The study by the University of Port Harcourt Teaching Hospital in Nigeria found that vacuum instrument types were significantly associated with maternal morbidity as forceps [21]. This variation could be the difference in operator competence and the choice of instrument with properly selected indications. But, the study conducted at the Jimma Medical Centre and Suhul County Hospital found that there is no difference in maternal complication in the two types of OVD used [13, 19]. This variation could be due to the sequential use of both types of operative vaginal delivery and equality of competence and expertise on both types of OVD.

Mothers whose second stage of labor was longer than 3 hours and who were assisted by a OVD had a significant association with the adverse mother outcome variable. However, a study conducted in the Stockholm/Gotland region of Sweden found that one and half hours of the second stage were at high risk after three hours or longer [20]. This variation could be the study area, the decision variety to conduct an OVD, and the intervention variety.

## 11. Conclusion

The magnitude of maternal complications of OVD is high. The types of instrument used for OVD, fetal head station, duration of second stage of labor, and neonatal birth weight were significantly associated with the maternal complications of OVD. Measures such as performing OVD with appropriate indications should be strengthened.

## Figures and Tables

**Table 1:** Sociodemographic characteristics of mothers who gave birth by OVD at NEMMCS hospital, Hadiya zone, southwest Ethiopia, 2021 (*n* = 326).

Variables		Frequency	Percent
Age (in years)	15–19	41	12.6
	20–24	108	33.1
	25–29	98	30.1
	30–34	35	10.7
	35–39	29	8.9
	>40	15	4.6

Marital status	Married	292	89.6
	Unmarried	34	10.4

Residence	Urban	262	80.4
	Rural	64	19.6

Occupation	House wife	147	45.1
	Farmer	101	31.0
	Civil servant	51	15.6
	Merchant	26	8.0
	Other	1	0.3

Religion	Muslim	59	18.1
	Orthodox	259	79.4
	Protestant	7	2.1
	Other	1	0.3

**Table 2:** Obstetric characteristics and maternal outcomes among mothers who gave birth by OVD at NEMMCS hospital, Hadiya zone, southwest Ethiopia, 2021 (*n* −  326).

Variables		Maternal complication		Total *n* (%)
		No *n* (%)	Yes *n* (%)	
Parity	1	151 (82.5%)	32 (17.5%)	183 (100.0%)
	2–4	106 (78.5%)	29 (21.5%)	135 (100.0%)
	≥5	7 (87.5%)	1 (12.5%)	8 (100.0%)

Gestational age	<37 weeks	26 (76.5%)	8 (23.5%)	34 (100.0%)
	37–42 weeks	210 (81.4%)	48 (18.6%)	258 (100.0%)
	>42 weeks	28 (82.4%)	6 (17.6%)	34 (100.0%)

Gestational age evidenced by	LNMP	186 (83.8%)	36 (16.2%)	222 (100.0%)
	Ultrasound	29 (78.4%)	8 (21.6%)	37 (100.0%)
	Fundal height	49 (74.2%)	17 (25.8%)	66 (100.0%)
	Other	0 (0.0%)	1 (100.0%)	1 (100.0%)

Number of ANC follow-up visits	No ANC visits	26 (86.7%)	4 (13.3%)	30 (100.0%)
	1–3 visits	86 (78.2%)	24 (21.8%)	110 (100.0%)
	4 or more visits	152 (81.7%)	34 (18.3%)	186 (100.0%)

Health institution where ANC attended	Primary hospital	109 (83.2%)	22 (16.8%)	131 (100.0%)
	NEMMCSH	51 (76.1%)	16 (23.9%)	67 (100.0%)
	Health center	68 (80.0%)	17 (20.0%)	85 (100.0%)
	Private medium clinic	10 (83.3%)	2 (16.7%)	12 (100.0%)
	Other	0 (0.0%)	1 (100.0%)	1 (100.0%)

Position of the fetal head	OA	132 (85.7%)	22 (14.3%)	154 (100.0%)
	OP	63 (68.5%)	29 (31.5%)	92 (100.0%)
	Unknown	69 (86.2%)	11 (13.8%)	80 (100.0%)

Episiotomy done	Yes	171 (85.1%)	30 (14.9%)	201 (100.0%)
	No	93 (74.4%)	32 (25.6%)	125 (100.0%)

Station during OVD application	Low	178 (76.7%)	54 (23.3%)	232 (100.0%)
	Outlet	86 (91.5%)	8 (8.5%)	94 (100.0%)

Duration of 2^nd^ stage of labor	1-<2 hrs	192 (84.2%)	36 (15.8%)	228 (100.0%)
	2-<3 hrs	46 (76.7%)	14 (23.3%)	60 (100.0%)
	≥3 hrs	26 (68.4%)	12 (31.6%)	38 (100.0%)

Weight of the newborn in gram	2500–3999 gm	188 (86.2%)	30 (13.8%)	218 (100.0%)
	1500–2499	23 (88.5%)	3 (11.5%)	26 (100.0%)
	≥4000 gm	53 (64.6%)	29 (35.4%)	82 (100.0%)

Types of OVD used	Vacuum	191 (85.3%)	33 (14.7%)	224 (100.0%)
	Forceps	73 (71.6%)	29 (28.4%)	102 (100.0%)

Previous place of delivery in the health institution	Yes	97 (78.2%)	27 (21.8%)	124 (100.0%)
	No	167 (82.7%)	35 (17.3%)	202 (100.0%)

Previous mode of delivery	SVD	59 (80.8%)	14 (19.2%)	73 (100.0%)
	OVD	29 (74.4%)	10 (25.6%)	39 (100.0%)
	C/s	9 (75.0%)	3 (25.0%)	12 (100.0%)

SVD, spontaneous vaginal delivery; OVD, operative vaginal delivery; C/s, cesarean section; ANC, antenatal care.

**Table 3:** Indications of OVD among mothers who gave birth by OVD NEMMCS hospital, Hadiya zone, southwest Ethiopia, 2019–2021 (*n* = 326).

Variables	Frequency		Percent
Prolonged 2^nd^ stage of labor	Yes	63	19.3
	No	263	80.7

Severe preeclampsia and/or eclampsia	Yes	16	4.9
	No	310	95.1

Heart disease	Yes	9	2.8
	No	317	97.2

Maternal exhaustion	Yes	45	13.8
	No	281	86.2

Severe anemia	Yes	10	3.1
	No	316	96.9

Fetal distress	Yes	183	56.1
	No	143	43.9

Malposition	Yes	89	27.3
	No	237	72.7

Others	Yes	1	0.3
	No	325	99.7

**Table 4:** Bivariate and multivariate logistic regression analysis of factors associated with the maternal outcomes of OVD at NEMMCS hospital, Hadiya zone, southwest Ethiopia, 2021.

Variables		Maternal complications		COR (95% CI)	AOR (95% CI)
		No *n* (%)	Yes *n* (%)		
Residence	Urban	40 (15.3%)	222 (84.7%)	1	1
	Rural^*∗*^	22 (34.4%)	42 (65.6%)	2.907 (1.570, 5.382)	1.177 (0.503, 2.755)

Position of the fetal head	OA	22 (14.3%)	132 (85.7%)	1	1
	OP^*∗*^	29 (31.5%)	63 (68.5%)	2.762 (1.471, 5.187)	1.821 (0.463, 7.168)
	Unknown	11 (13.8%)	69 (86.2%)	0.957 (0.438, 2.087)	1.509 (0.606, 3.755)

Episiotomy for instrument application	Yes^*∗*^	30 (14.9%)	171 (85.1%)	0.510 (0.292, 0.891)	0.624 (0.329, 1.185)
	No	32 (25.6%)	93 (74.4%)	1	1

Station during OVD used	Low^*∗∗*^	54 (23.3%)	178 (76.7%)	3.261 (1.486, 7.156)	3.199 (1.359, 7.533)
	Outlet	8 (8.5%)	86 (91.5%)	1	1

Duration of 2^nd^ stage of labor	1-<2 hrs	36 (15.8%)	192 (84.2%)	1	1
	2-<3 hrs	14 (23.3%)	46 (76.7%)	1.623 (0.809, 3.256)	1.661 (0.714, 3.865)
	≥3^*∗∗*^	12 (31.6%)	26 (68.4%)	2.462 (1.138, 5.322)	2.556 (1.039, 6.284)

Neonatal weight (in gm)	2500–3999 gm	30 (13.8%)	188 (86.2%)	1	1
	1500–2499	3 (11.5%)	23 (88.5%)	0.817 (0.231, 2.891)	0.543 (0.122, 2.425)
	≥4000 gm^*∗∗*^	29 (35.4%)	53 (64.6%)	3.429 (1.892, 6.214)	3.342 (1.435, 7.787)

Type of OVD used	Vacuum	33 (14.7%)	191 (85.3%)	1	1
	Forceps^*∗∗*^	29 (28.4%)	73 (71.6%)	2.299 (1.304, 4.054)	2.248 (1.144, 4.416)

Prolonged 2^nd^ stage of labor	Yes^*∗*^	19 (30.2%)	44 (69.8%)	2.209 (1.177, 4.146)	0.901 (0.399, 2.035)
	No	43 (16.3%)	220 (83.7%)	1	1

Fetal distress	Yes^*∗*^	24 (13.1%)	159 (86.9%)	0.4171 (0.236, 0.736)	0.608 (0.305, 1.212)
	No	38 (26.6%)	105 (73.4%)	1	1

Mal position	Yes^*∗*^	28 (31.5%)	61 (68.5%)	2.741 (1.540, 4.877)	1.559 (0.392, 6.205)
	No	34 (14.3%)	203 (85.7%)	1	1

^*∗*^ *p* value set at ≤0.2 for binary logistic regression; ^*∗∗*^*p* value set at 0.05 for multilogistic regression.

## Data Availability

The data used is available from the author upon request.

## References

[1] Coombes R. (2008). Healthcare Commission will publicise NHS trusts’ levels of infection control. *BMJ*.

[2] Gabbe S. G., Niebyl J. R. (2012). *JLS at Al. Instrumental Delivery*.

[3] Mcquivey R. W. (2004). Vacuum-assisted delivery. *Journal of Maternal-Fetal and Neonatal Medicine*.

[4] Prameela R. C., Asha M. B. S. P. (2015). Outcome of instrumental vaginal deliveries in referred cases. *JEMDS*.

[5] Ali U. A., Norwitz E. R. (2009). Vacuum-assisted vaginal delivery. *Rev Obstet Gynecol. Winter*.

[6] Berhan Y., Abdela A., Berhan Y., Ethiopian A. A. (2004). Emergency obstetric performance with emphasis on operative delivery outcome. *Journal of Health Development*.

[7] Alegbeleye J. O., Orazulike N. C., Nyengidiki T. K., Uzoigwe S. A. (2018). Instrumental vaginal delivery at the university of Port Harcourt teaching hospital, Port Harcourt, Nigeria. *Tropical Journal of Obstetrics and Gynaecology*.

[8] Fatima A. (2014). Comparison of maternal and neonatal outcome in forceps versus ventouse assisted vaginal delivery. *Annals of Punjab Medical College (APMC)*.

[9] Dietz H. P., Lanzarone V., Simpson J. M. (2016). Predicting operative delivery. *Ultrasound in Obstetrics and Gynecology*.

[10] Factors I. A., Undergone W., Delivery O. (2018). Prevalence and its associated factors among women undergone operative delivery at Hawassa university comprehensive specialized gynecology & Obstetrics. *Gynecology & Obstetrics*.

[11] Aiken C., Aiken A. R., Brockelsby J. C. (2014). Feto Factors Influencing the Likelihood of Instrumental Delivery Successaternal outcomes and associated factors of instrumental delivery. *Obstetrics & Gynecology*.

[12] Shamsa A., Bai J., Raviraj P., Gyaneshwar R. (2013). *Types of OVD and its Associated Maternal and Neonatal Outcomes*.

[13] Gebre S. H. A. (2017). Complications of instrumental vaginal deliveries and associated factors at shul genaral hospital. *Tigriya*.

[14] Faisal S., Bava A., Nandanwar Y. S. (2016). Instrumental vaginal deliveries at tertiary centre. *J Reprod Contracept Obs Gynecol*.

[15] Article O. (2013). Comparison of maternal and fetal outcome in instrumental delivery: vacuum versus forceps vaginal delivery. *The Journal of Obstetrics and Gynecology of India*.

[16] Jabeen N., Baloch R., Malhi P., Zahiruddin S., Mawani K. (2017). Foeto-maternal outcome in instrumental vaginal delivery attending a secondary hospital in Hyderabad (aga khan maternal and child care centre). *Journal of Pakistan Medical Association*.

[17] Prabha A., Anita S., Kate D. (2010). Instrumental delivery. *Journal of Obstetrics and Gynaecology (Basingstoke)*.

[18] Shrestha B. K., Shrestha S. (2016). Short term maternal and neonatal outcome in a tertiary hospital of Nepal. *IJRCOG*.

[19] Hubena Z., Workneh A., Siraneh Y. (2018). Prevalence and Outcome of Operative Vaginal Jimma University Medical Center, Southwest Ethiopia. *J Pregnancy*.

[20] Simic M., Cnattingius S., Petersson G., Sandström A., Stephansson O. (2017). Duration of second stage of labor and instrumental delivery as risk factors for severe perineal lacerations: population- based study. *BMC Pregnancy Childbirth*.

[21] Alegbeleye J. O., Orazulike N. C., Nyengidiki T. K., Uzoigwe S. A. (2018). Original Article A 10-Year Review of Instrumental Vaginal Delivery at the University of Port Harcourt Teaching Hospital, Port Harcourt. *Tropical Journal of Obstetrics and Gynaecology*.

